# Risk factors for asthma

**DOI:** 10.1186/1824-7288-40-S1-A77

**Published:** 2014-08-11

**Authors:** Michele Miraglia del Giudice, Annalisa Allegorico, Giuseppe Parisi, Francesca Galdo, Emilia Alterio, Amalia Coronella, Giuseppina Campana, Cristiana Indolfi, Natja Valenti, Sonia Di Prisco, Serena Caggiano, Nunzia Maiello

**Affiliations:** 1 Department of women and child and general and specialized surgery, Second University of Naples, Italy; 2 Rizzoli Hospital of Ischia, Naples, Italy

## 

Asthma is the most common chronic respiratory disease of childhood, and even if there have been many advances in the understanding pathogenesis of the disease, many aspects remain to be clarified.

In the pathogenesis of asthma are involved both "protective" and "predisposing" factors as a result of the complex interactions that occur between genetic predisposition and environmental exposure.

From the genetic point of view, the identified genes responsible are more than 100, and many polymorphisms have been shown to be associated to the onset of asthma, although none of these, alone or in combination, is able to predict the occurrence of disease.

The environmental factors most involved in the onset of asthma in children are represented by allergens, tobacco smoke, respiratory infections and air pollution.

Indoor allergens (dust mites, mold and animal dander) and outdoor (pollens and molds) are able to induce sensitization by prolonged exposure and trigger acute asthma. Allergic sensitization, in the concept of atopic march, represents a major risk factor for the development of asthma. In particular, the subjects polysensitized and with food allergy may present more severe asthma [1].

The exposure to cigarette smoke in both prenatal and postnatal increases the risk of the child becoming asthmatic and the asthma severity.

It has also noted recently that obesity is a risk factor for asthma because it causes an increase of leptin, TNF-α, and IL-6, which exert a pro-inflammatory non-eosinophil action [2]. In addition, the lack of physical activity, for weight gain, contributes to the determinism of the disease [3].

Vitamin D is involved in the processes of development and fetal lung maturation; the levels of 25-OH vitamin D from umbilical cord blood are inversely correlated with the risk of respiratory infections and wheezing in childhood [4]. The vitamin D has immunomodulatory properties exerting an action of inhibiting the production of pro-inflammatory cytokines and induction of the synthesis of antimicrobial peptide on cells of the innate immune system [5]. The vitamin D modulates the effects of glucocorticoids and also has a role in bronchial remodeling, as it regulates the expression of genes of bronchial smooth muscle.

Infections early in life may play a role of "induction" of wheezing or "protection" against the development of allergic diseases (according to the hygiene hypothesis)[6-8]. In infants at risk viral respiratory infections can cause wheezing, which in turn can evolve later in asthma particularly in individuals with atopic predisposition.
